# Spatial distributions of Pseudomonas fluorescens colony variants in mixed-culture biofilms

**DOI:** 10.1186/1471-2180-13-175

**Published:** 2013-07-28

**Authors:** Matthew L Workentine, Siyuan Wang, Howard Ceri, Raymond J Turner

**Affiliations:** 1 Biofilm Research Group, Department of Biological Sciences, University of Calgary, 2500 University Dr NW, AB, T2N 1N4, Calgary, Canada; 2 Department of Medicine, McMaster University, 1200 Main St W, ON, L8S 4K1, Hamilton, Canada

## Abstract

**Background:**

The emergence of colony morphology variants in structured environments is being recognized as important to both niche specialization and stress tolerance. *Pseudomonas fluorescens* demonstrates diversity in both its natural environment, the rhizosphere, and in laboratory grown biofilms. Sub-populations of these variants within a biofilm have been suggested as important contributors to antimicrobial stress tolerance given their altered susceptibility to various agents. As such it is of interest to determine how these variants might be distributed in the biofilm environment.

**Results:**

Here we present an analysis of the spatial distribution of *Pseudomonas fluorescens* colony morphology variants in mixed-culture biofilms with the wildtype phenotype. These findings reveal that two variant colony morphotypes demonstrate a significant growth advantage over the wildtype morphotype in the biofilm environment. The two variant morphotypes out-grew the wildtype across the entire biofilm and this occurred within 24 h and was maintained through to 96 h. This competitive advantage was not observed in homogeneous broth culture.

**Conclusions:**

The significant advantage that the variants demonstrate in biofilm colonization over the wildtype denotes the importance of this phenotype in structured environments.

## Background

When grown in spatially structured environments several *Pseudomonas* species are known to produce variants with altered phenotypic properties. Such variants are often isolated from laboratory biofilms [1-5], cystic fibrosis airways [6,7], and the plant rhizosphere [8]. Two variant types have been characterized in some detail; the wrinkly spreader (WS, also called rugose small colony variants) and the small colony variant (SCV), of which the primary phenotypic characteristic is the overproduction of exopolyscharides [1,2,6,9].

Given that these variants arise in structurally heterogeneous environments, presumably still populated with the ancestral strain, one could expect the variants to have an advantage in specific niches within these environments. Indeed, the WS morphotype isolated from static microcosms has a competitive advantage at the air-liquid interface where it can form self-supporting mats generated by the cellulose-like polymer that it overproduces [1,10-12]. However, besides competition studies with this morphotype very little work has been done to examine spatial interaction between colony variants and the ancestral phenotype, within the environment where the variant evolved. To the best of our knowledge only one other study has specifically examined the spatial distributions of variant and wildtype populations in a biofilm on a microscopic level. This was done with a laboratory derived *P. aeruginosa* colony variant and the authors concluded that the variant only had a selective advantage in certain niches within the biofilm [4].

We have previously isolated SCV and WS variants from biofilms of *P. fluorescens*[2]. To examine spatial interactions between colony variants and the wildtype ancestral strains, strains were labeled with 4 different coloured auto-fluorescent proteins (AFPs). In order to determine if these variants had any spatial preference or advantage in the environment where they evolved we examined co-culture biofilms and planktonic populations of SCV and WS with the ancestral strains.

## Results and discussion

The emergence of phenotypic diversity in biofilms or other structurally heterogeneous environments is generally associated with selection for that phenotype in that particular environment. Such is the case for the previously studied WS from *P. fluorescens* SBW25, which has adaptations that allow it to out-compete wildtype genotypes from the air-liquid interface of the static microcosm where it evolved [1]. Previously we isolated an SCV and WS variant from a *Δ*gacS strain of *P. fluorescens* biofilms and here we sought to determine if these variants might have an advantage in the biofilm environment. The hypothesis was that the variants would have a distinct advantage over the wildtype, when colonizing a surface, due to the fact that they evolved in the biofilm. In addition, the fact that the WS is over-producing a cellulose-like polymer [2] suggests it might be better at colonizing a surface.

To test this hypothesis, different coloured auto-fluorescent proteins (AFPs) were introduced into the four different strains of *P. fluorescens*; CHA0 (wildtype), CHA19 (*Δ*gacS), SCV, and WS. The two variants were isolated from the *Δ*gacS strain, which produces a higher frequency of colony morphology variants [2] and so both the wildtype and *Δ*gacS strains were included. Green fluorescent protein (GFP), yellow fluorescent protein (YFP), cyan fluorescent protein (CFP), and dsRed (referred to from here on in as red fluorescent protein, RFP) were introduced on a plasmid that is stable in *P. fluorescens* without antibiotic selection [13]. Biofilms of the individual strains or mixed co-cultures were grown and imaged using confocal laser scanning microscopy (CLSM). Imaging the individual strains with each of the 4 colours of AFP revealed that expressing the different fluorescent proteins did not significantly alter the biofilm structure when compared to the biofilms stained with acridine orange [2]. Although some variation in biofilm structure was observed between replicates, this was independent of which AFP was being expressed, indicating that no one particular AFP was affecting biofilm formation or structure.

**Figure 1:**
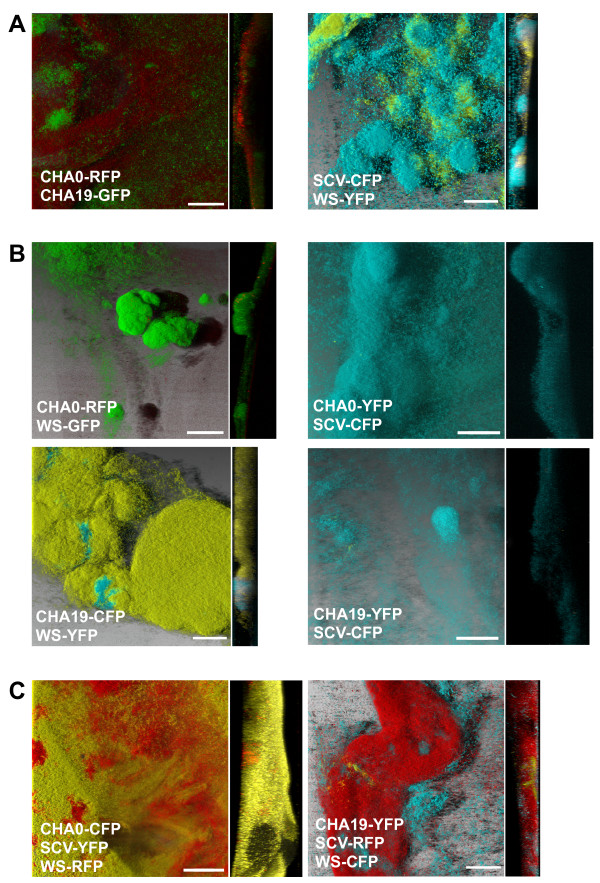
**Analysis of variant and ancestral strain biofilm co-cultures.***P. fluorescens* variants and ancestral strain co-cultures were analyzed by the introduction of different colour AFPs. CLSM images were obtained on 96 h biofilms grown in the CBD. See ‘Materials and Methods’ for details of acquisition parameters. Multiple replicates were obtained for each biofilm co-culture and shown here are the best representative images. The images show a top-view 3D reconstruction of the biofilm along with a cross-section through the y-axis. Scale bars represent 40 *μm*. **A**, Controls showing that the two variants grow evenly together and the wildtype (CHA0) and *Δ*gacS (CHA19) also grow evenly distributed throughout the biofilm. **B**, Paired co-cultures of the CHA0 and CHA19 strains with either the SCV or WS. **C**, Triple co-cultures were done, where the SCV and WS were cultured together with either CHA0 or CHA19.

**Figure 2:**
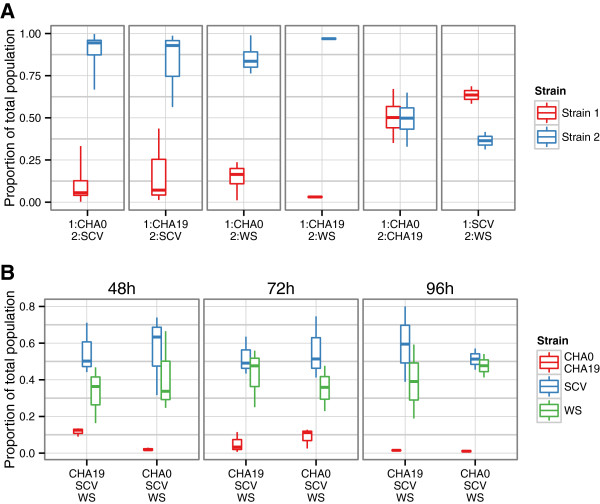
**Quantification of biomass in biofilm co-cultures.** The amount of each strain in the biofilm was quantified from multiple images. Shown is the relative proportion of each strain in the total population. **A**. Pair-wise comparisons of different strain combinations at a single time point. **B**. Quantification of the time-course images where three strains were used in each co-culture.

For the initial analysis a pair-wise matrix was setup, whereby each strain was co-cultured with each of the other strains and this was performed with two pairs of AFPs, a GFP-RFP pair and a CFP-YFP pair. In all cases a further control was performed where the protein pairs were reversed between strains. Both of these controls ensured that variations in expression between the different plasmids would be accounted for. Representative images from multiple growth replicates (at least 3) are shown in Figure 1 and quantification of these images is shown in Figure 2. When CHA0 is co-cultured with the *ΔgacS* the two strains are distributed evenly throughout the biofilm and neither one appears to overgrow the other (Figure 1A and 2A) (*p*=0.90). This is also the case when the SCV and WS are cultured together (*p*=0.07), although the SCV may have a slight advantage over the WS (Figure 2). However, when either the SCV or WS are cultured with CHA0 or CHA19, the variant appears to almost completely out-compete the parental strains (*p*<0.02 for all pairwise comparisons). As can be seen in Figure 1B there are only small patches of CHA0 or CHA19 in biofilms dominated by the SCV or WS. In some cases no CHA0 or CHA19 cells were visible in the image.

**Figure 3:**
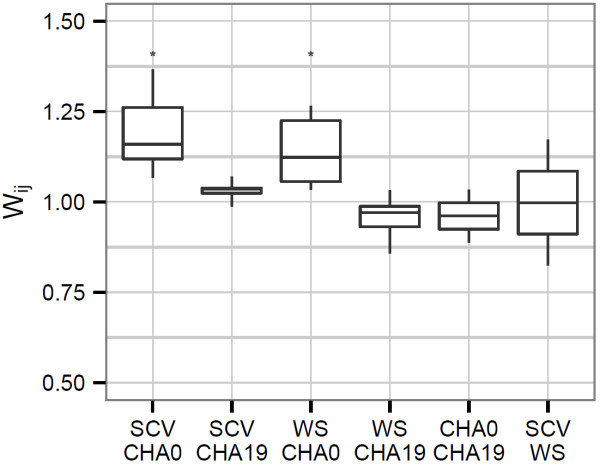
**Relative fitness of the variants when co-cultured in shaken tubes with the wildtype parental strains.** A value above 1 indicates the variant has a competitive advantage over the parental strain. The asterisk indicates a mean fitness that is significantly higher than 1 (*p*<0.05).

In contrast when the strains were competed in shaking planktonic culture there was little to no competitive advantage of the variants over the wildtype strains (Figure 3). The WS and SCV did have an advantage over the CHA0 strain (*p*=0.048 and 0.027, respectively), however the relative fitness values were low indicating that CHA0 still made up a large proportion of the population unlike what was seen with the biofilm cultures. Final cell densities of the two strains differed by less than 0.5 logs.

**Figure 4:**
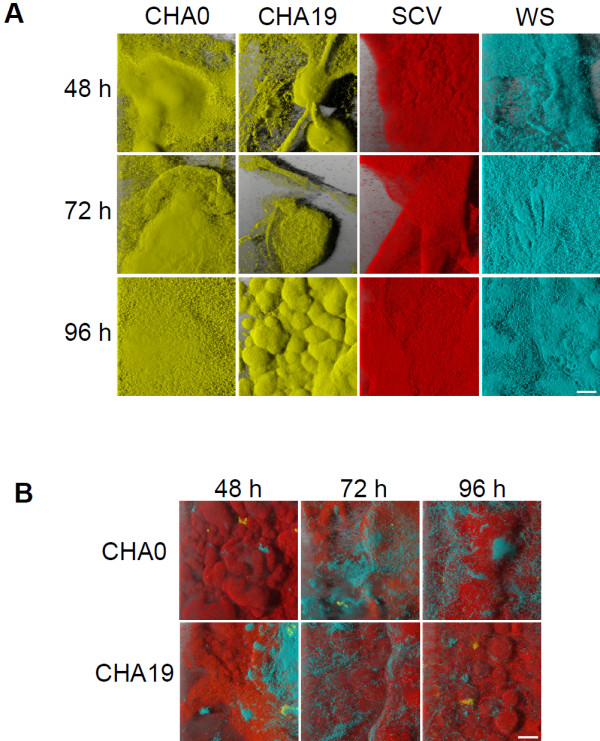
**Time-course analysis of variant and wildtype population distributions in biofilms.** A time-course of the individual populations of CHA0, CHA19, SCV, and WS **(A)**, and the SCV and WS in mixed co-culture with either CHA0 or CHA19 **(B)**, was done over a period of 96 h to determine how quickly the variant populations were overtaking the biofilm. CHA0 and CHA19 are expressing YFP, SCV is expressing RFP and the WS is expressing CFP. The images shown are 3D reconstructions from the CLSM z-stacks and each image was acquired from a separate peg in the CBD. All images were acquired at the same resolution and scale bars in the bottom right of each panel represent 40 *μm*.

Co-culture experiments were also done where both the SCV and WS were cultured together along with either CHA0 or CHA19. The results from the triple co-culture are shown in Figure 1C and demonstrate a similar result as the paired analysis with the two variants being evenly distributed but very little CHA0 or CHA19 cells in the biofilm. The triple co-cultures were then used for a time course experiment to determine if the parental strains were co-colonizing the surface with the variants and then being out-competed in a mature biofilm or if the WS and SCV were colonizing the surface better and excluding the parental strains. Images of the strains grown individually were acquired at various time points throughout a total growth time of 96 h. In all cases the individual populations were able to efficiently colonize the peg surface (Figure 4A). However, within 48 h of inoculation the two variants already made up the majority of the biofilm with this trend continuing at the remaining time points (Figure 4B and 2B). This suggests that the two variants are better able to colonize the surface of the peg, thereby excluding the parental strains who, when grown individually are capable of forming substantial biofilms.

Niche specialization is an important aspect of colony morphotypes and this is certainly the case for the variants described in this study. Here we have shown that the SCV and WS colony variants out-grow the ancestral populations in the environment from which they were isolated, that is, the peg surface in the CBD. Microscopic evaluation of spatial distributions of variant and ancestral strains in biofilms is virtually non-existent, hence, these findings represent the first detailed microscopic examination of multiple variant types within a biofilm. One previous study examined a variant and wildtype co-culture of *P. aeruginosa* in a tube biofilm [4]. Here they observed that although the variant seemed to dominate initially, upon prolonged growth the wildtype eventually took over and the variant never made up more than 40% of the biofilm. The conclusion was the variant was only able to grow within certain microniches in the tube biofilm. Given the microscale heterogeneity assumed to be present in the biofilm environment [14] such microniche specialization could certainly be expected. However, the work here suggests that, at least for *P. fluorescens*, the two morphotypes are macroniche specialists, that is, they have adaptations that allow them to better colonize the entire surface, rather than small niches within the biofilm. The extensive work done with the WS morphotype from *P. fluorescens* SBW25 supports this concept in that this morphotype is adapted to colonize the air-liquid interface of static microcosms, a niche that cannot be colonized by the wildtype phenotype [1].

It is interesting to note that in the present study, the wildtype can colonize the peg surface efficiently suggesting that the emergence of diversity is not solely associated with ecological opportunity but may have other function such as resistance to stress, as is suggested by the enhanced metal tolerance these variants have over the ancestral *ΔgacS* strain [2]. In addition to having properties suggestive of adaptation to surface growth variants of *P. aeruginosa* isolated from the lungs of infected cystic fibrosis patients also have markedly increased antibiotic resistance [6]. This has lead to the general conclusion that these variants have more than just surface-attachment adaptations but may actually have a host of adaptations specific to the environment from which they were isolated [5].

## Conclusions

In summary, we have presented a microscopic examination of variant-wildtype distributions in biofilms, which has revealed that the variants rapidly out-grow the wildtype and dominate the biofilm environment. Furthermore, we demonstrate that this is phenomenon is specific to surface associated growth and is not observed in planktonic culture.

## Methods

### Bacterial growth

All strains (Table 1) were routinely cultured on LB agar or LB media at 30℃, stored at -80℃ in MicroBank^™^ vials and sub-cultured no more than twice prior to experimentation. The plasmids expressing the different coloured AFPs were introduced into *P. fluorescens* by electroporation according to previous protocols [15]. The colony variants (WS and SCV) were derived from the *ΔgacS* strain which produces phenotypic variants when exposed to heavy metal stress [2]. Introduction of the plasmids had no observable effects on colony morphology. Biofilms were cultured in LB using the Calgary Biofilm Device (CBD) [16,17], with shaking at 150 rpm, at 30℃ and approximately 95% relative humidity. A 1:30 dilution of a 1.0 McFarland standard was prepared for each individual strain and the CBD was inoculated with either the individual strain or a 1:1 mixture of the two or three strains being co-cultured and then grown for the indicated time prior to imaging. Due to the extended growth times for this experiment (up to 96 h) viable cell counts could not be obtained as the *P. fluorescens* variants grow very thick biofilms that could not be entirely removed by sonication. No new phenotypes were observed after 96 h of growth with any of the strains.

**Table 1:** Strains and plasmids used in this study

**Strain or plasmid**	**Description**	**Source**
*P. fluorescens* CHA0	Wild-type strain	[18]
*P. fluorescens* CHA19	Contains a marker-less deletion of the *gacS* coding region	[18]
*P. fluorescens* SCV	Small Colony Variant derived from the CHA19 strain	[2]
*P. fluorescens* WS	Wrinkly Spreader derived from the CHA19 strain	[2]
pME6010	Rhizosphere stable plasmid, does not require antibiotic selection in *P. fluorescens*	[19]
pMP4655	pME6010 containing the coding sequence of enhanced GFP with the *lac* promoter	[13]
pMP4641	pME6010 containing the coding sequence of enhanced CFP with the *lac* promoter	[13]
pMP4658	pME6010 containing the coding sequence of enhanced YFP with the l*ac* promoter	[13]
pMP4662	pME6010 containing the coding sequence of dsRed with the *lac* promoter	[13]

### Microscopy and biofilm quantification

Microscopy was performed according the protocols outlined previously [20]. The pegs were examined using a Leica DM IRE2 spectral confocal and multiphoton microscope with a Leica TCS SP2 acoustic optical beam splitter (AOBS) (Leica Microsystems). A 63 × water immersion objective used for all the imaging and the image capture was performed using Leica Confocal Software Lite (LCS Lite, Leica Microsystems). Imaging of the biofilms expressing the AFPs were obtained by breaking off a peg of the CBD and placing it on a coverslip with a drop of saline. Excitation/emission parameters for each of the AFPs were 488/500−600 for GFP, 514/525−600 for YFP, 458/465−600 for CFP, and 543/55−700 for dsRed. To reduce cross-talk between the different AFPs, images with more than one AFP were acquired sequentially by frame so only one AFP was being imaged at a time. Furthermore any AFPs that were imaged together were checked to ensure minimal cross-talk was occurring. Laser intensity and photomultiplier tube gain were kept consistent across all experiments. Image stacks were processed using Imaris 6.3.1 (Bitplane) to generate images for publication. Biovolumes for each image stack were computed using the ‘Surfaces’ feature of the Imaris software with the ‘Absolute Intensity’ setting for background removal. For each co-culutre, 4 replicates comprised of different strain-AFP combinations (to remove any fluorescent intensity bias in the quantification) were used to calculate the mean biovolume. The relative proportion of each strain was calculated compared to the total biovolume. Student’s t-test was used to compare the means of the relative volumes for each strain pair.

### Planktonic competition

To determine if the WS or SCV had any growth advantage in broth culture competitions were performed with each pair combination. Equal volumes of 16 h cultures of each strain were add to a total of 150 *μ*L LB media in 96 well plates (30-fold dilution). The plate was incubated at 30℃ with shaking (175 rpm) for 24 h. Prior to incubation samples were removed for determination of initial cell numbers. The cultures were serially diluted on LB agar and the number of each colony type were recorded. The SCV and WS could easily be distinguished from the wildtype CHA0 and CHA19 colony types. To control for any phenotypic variation occurring the broth culture the competitions were performed with the strains expressing the fluorescent proteins. Representative plates from each pair combination were imaged with a fluorescent imager (IVIS Imaging System, Caliper LifeSciences) to distinguish the two strains and the numbers were compared to the values obtained when counting based on colony morphology. No phenotypic variation occurred in broth cultures during the time period tested. Fluorescent imaging of the plates was also used to distinguish the CHA0 and CHA19 colonies as well as CHA0 and CHA19 competed with themselves. The relative fitness [21] of the variant (SCV or WS) compared to wildtype (CHA0 or CHA19) was calculated for each pairwise combination. A relative fitness of 1 indicates that neither strain has a competitive advantage, whereas values higher than 1 indicate that the variant is more fit in the broth culture. A one-tailed Student’s t-test was used to determine if the values were significantly greater than 1. P values were adjusted with the Holm-Bonfferoni correction to control for the family-wise error rate [22].

## Competing interests

The authors declare no competing interests.

## Authors’ contributions

MLW and RJT designed the study and wrote the manuscript. MLW performed the experimental work with assistance from SW. HC assisted with study design and data interpretation. All authors read and approved the final manuscript.
